# Effectiveness of rural internships for veterinary students to combat veterinary workforce shortages in rural areas

**DOI:** 10.1371/journal.pone.0294651

**Published:** 2024-03-07

**Authors:** Mehdi Berrada, Didier Raboisson, Guillaume Lhermie

**Affiliations:** 1 CIRAD, UMR ASTRE, Montpellier, France; 2 ASTRE, CIRAD, INRAE, Univ Montpellier, Montpellier, France; 3 ENVT, Université de Toulouse, Toulouse, France; 4 Faculty of Veterinary Medicine, University of Calgary, Calgary, Canada; University of Agriculture Faisalabad, PAKISTAN

## Abstract

Veterinarians are a pivotal force in addressing animal health and welfare surveillance, with a critical role in improving public health security and increasing the profits of livestock farmers. Yet, the veterinary profession is adversely affected by personnel shortages, particularly in rural areas. Since the health of people, animals and their shared environment are interconnected in a One Health perspective, a set of policies are required to ensure public health by attraction and retention of veterinarians in rural areas. In France, a tutored internship programme, financially subsiding students and mentors to execute a training period in remote rural areas, was promoted to better integrate and retain veterinary students ending their veterinary training. This paper aims to evaluate how veterinarians’ tutored internships influences students’ choices for rural practice, using three different statistical methods derived from causal inference theory. Using survey data for the period 2016–2020, we show that: (i) the average effect of the tutored internship on veterinarians’ work in food animal sector is not significant; and that (ii) the tutored internship leads veterinarians with a low share of work in the food animal sector to have a rural practise after they graduated between 13 and 20% greater than those who did not participate in the tutored internship.

## 1. Introduction

A shortage of veterinarians is a major concern for the profession, particularly in rural remote areas. In this paper, the term “shortage” is not used in the economic sense, it indicates a lack of sufficient veterinarians’ supply to cover demand needs. Between 2016 and 2020, the percentage of veterinarians in the food animal sector (FAS) plummeted by 18% in France [1]. The definition and location of a veterinarian shortage in France has been mapped, enabling public and private authorities to establish appropriate measures to address this concern, as done for physician shortage [2]. France is not unique when it comes to a veterinarian shortage. In Europe, the Federation of Veterinarians of Europe (FVE) conducted a survey on the veterinarian shortage in rural areas in 2020 [3]. Among 28 European countries surveyed, 78.5% are experiencing a shortage of veterinarians. In 2017, a survey was conducted in 11 EU countries to assess the veterinarian shortage of their territory by rural veterinarians. Abandonment of rural areas by the veterinary profession in these countries was described as alarming and raising the question of food security [4]. At the Group of Eight (G8) Summit in Italy, in July 2009, it was put forward to “act with the scale and urgency needed to achieve sustainable global food security.” The challenge of global food security requires a huge effort where veterinarians play a pivotal role in overcoming food insecurity, dealing with the devasting effects of infectious animal diseases and alleviating zoonotic spillovers and improving the economic situation of small livestock farmers.

Policymakers seeking to address the veterinary shortage offered many solutions. The U.S. is also facing a shortage of veterinarians, especially those who have food animal (FA), agricultural and public health practice experience [5]. It was revealed that 16 of 50 states had a shortage of rural veterinarians [6].

In the U.S., the National Veterinary Medical Service Act was adopted in 2003 to authorize the Secretary of Agriculture to conduct a loan repayment programme for veterinary services experiencing a shortage situation [7], with the final aim of encouraging veterinarians to practice in rural areas. In addition, the Veterinary Public Health Workforce Expansion Act of 2005 was established to award competitive grants to eligible entities to increase the number of veterinarians. To promote recruitment of veterinarians, the U.S. Department of Agriculture (USDA) has also two programmes: The Veterinary Medicine Loan Repayment Programme and Veterinary Services Grant Programme reference. This federal programme provides loan repayment assistance to animal and public health veterinarians who commit to practising for at least 3 years in veterinary shortage areas identified by state veterinarians and the USDA, providing them the financial assistance they need to embark on a rural veterinary career [8].

In France, a set of measures and incentives to tackle veterinary shortages have been implemented that aim to target graduate veterinarians and decrease attrition. Among these measures, one can mention the increase in the number of students by modification of the selection criteria in recruitment procedures in National Veterinary Schools [1], ‘VETER’ internships and ‘Terre d’Accueil’ project, tutored internships [9], tax reliefs in rural areas [10], grant support for the start-up of young veterinarians [11] and an opportunity for veterinarians to combine work and pension receipts [12].

### 1.1 Related literature

To date, there are no studies evaluating the effectiveness of programmes aiming at retaining veterinarians work in rural areas. In contrast, physician supply retention in underserved areas has long been an interest of health and labor economists [13–15]. Since physician shortage in rural rural areas represents a similar situation than veterinarian shortage, we will draw results from the literature in human medicine. Hurley analysed physicians location choice and demonstrated that income and the community size are significant factors in the location choice among physicians [16]. Bolduc examined the distribution of physicians in Québec and provided evidence that incentives in incomes had a significant effect on physicians’ location choices [17]. Other research measured the effectiveness of retention program as the National Health Service Corps (NHSC) program, which is a United States program charged with increasing physician supply and equilibrate geographical access to primary health care [18]. Holmes found that deleting this program would decrease the supply of physicians in medically underserved communities by roughly 10%.

### 1.2 Description of the tutored internship programme

Tutored internships were promoted by the French Senate which adopted a bill to address the veterinary shortage in 2013. Between 2013 and 2015, tutored internships were still in the experimental stage and only few students have taken part in it. This public policy aims to better integrate students who are in their fifth and final year into rural areas. The tutored internship concerns only students who have a fifth year with FA focus, and not other students who have chosen to pursue the companion animal sector. This internship is optional, takes place during a semester of study in a rural environment, and lasts from 18 weeks to 6 months in one or exceptionally two veterinary practices/clinics with significant rural activity. Only veterinary practices/clinics that meet requirements are eligible to train a student as a part of a tutored internship. These are then subject to an accreditation by a steering committee from the NVSs (National Veterinary Schools). The aim of this programme is threefold: (i) improve clinical skills; (ii) understand practice management of a clinic and clients’ relationship; and (iii) explore lifestyle and practising veterinary medicine in rural areas. All of this is conducted under the supervision of the veterinary schools’ teaching team. This measure has steadily increased over time, and mainly since the 2016–2017 school year, when financial support from the French ministry of Agriculture - DGAL (Direction Générale de l’Alimentation) was provided to cover all related expenses. In 7 years, this scheme has involved 214 students across the 4 French NVSs. Between 2016 and 2021, 36% of the mentees were from NVS of Toulouse, 28% from NVS of Lyon, 22% from NVS of Nantes and 14% from NVS of Alfort. As the tutored internship was only in its test phase between 2013 and 2016, we have only measured impacts of the tutored internship after 2016.

By estimating effects of the tutored internships on veterinarians’ attitudes with regards to their career choices, this study provides policy makers key information to decide whether the programme should be stopped or sustained.

### 1.3 Conceptual model: Programme evaluation

Many countries have shortages of health care personnel [19] and invest heavily in programmes or policies. Economists have the role of evaluating these programmes in order to measure and improve their effectiveness [20]. In the last decade, numerous policy evaluations using causal inference methods, whether experimental or quasi-experimental, have been applied by economists to various topics such as education [21], agriculture [22, 23], consumer credit markets [24] or labour market policies [25, 26]. These methods are based on observation schemes which ensure that estimated differences in outcomes are mainly due to the intervention or policy implemented and that selection biases are reduced or eliminated. To know the gain from a programme for each person, one computes the difference between the participants’ outcome with and without the treatment i.e., the policy implemented. However, it is impossible to observe these two situations simultaneously for a given participant. Thus, causal inference methods aim to statistically identify the counterfactual outcome i.e., what would have been the outcome of participants to a programme if the intervention was implemented. Therefore, a fundamental evaluation problem arises here: given some treated individuals, how can we estimate what would be the outcome of these individuals if they were not treated?

Experimental methods consist of randomly selecting participants to a programme to be evaluated and comparing their outcome with that of non-participants. They demonstrate the causal link between a public policy and the observed effects without restrictive assumptions. Experimental methods, as the Randomized Controlled Trial, are considered the gold standard in terms of estimating the causal effect of treatment. However, in practice, many experimental studies are not randomized due to economic and temporal reasons, or for ethical reasons. In this case, so-called quasi-experimental methods are used. They seek to identify situations where participation in a program is independent of the characteristics of the units targeted by this programme [27]. Among the quasi-experimental methods, matching techniques evaluate effects of a programme by pairing participants to a programme with members of a control group who have similar pre-treatment characteristics. That being done, programme impact is estimated by subtracting mean outcomes of matched control group members from mean outcomes of matched participants.

In this study, a program evaluation using matching methods was conducted to estimate effects of veterinarians’ participation in a tutored internship on their propensity to work in the food animal sector.

## 2. Materials and methods

### 2.1 Survey

A questionnaire was carried out between November 1^st^ and 30, 2022 and sent to veterinarians of the 4 French NVSs, who chose a fifth year with a FA focus. Before starting the questionnaire, participants were informed of the purpose and the description of the study and how the data collected would be used, anonymized and deleted once the study was completed. If the participant accepted these conditions, he or she could start the questionnaire and had the right to withdraw. The ethics committee (Comité d’Ethique de Recherche de l’Université de Toulouse) that approved this study confirmed that clear information provided to participants was sufficient, and that there was no need for written or oral consent.

All participants answered a set of questions constituting pre-treatment observable characteristics. Once the answers were collected, two groups were distinguished: the treatment group, those who chose a fifth year with a FA focus and participated in the tutored internship, and the control group, those who chose the same fifth year but did not participate in the tutored internship. We distinguished between these two groups because veterinarians who chose a fifth year with a FA focus but did not participate in the tutored internship had the same courses and similar characteristics to those who chose the tutored internship; therefore, this group represents a relevant control group.

This questionnaire was designed to obtain data on characteristics of the veterinarians (covariates) that will serve to match the two groups, whether they followed a tutored internship (treatment variable) and the share of work in the FA sector (outcome variable).

All questions are summarised in S1 Table.

### 2.2 Programme evaluation

#### 2.2.1 Measure of programme effects

Evaluation studies attempt to estimate effects of participating in a programme called treatment. This requires estimating a counterfactual outcome i.e., outcome that would have been observed for the treated if they had not been treated. The treatment group in this study is the veterinarians who participated in the tutored internship, whereas the control group is veterinarians who chose a fifth year with a FA focus and who were eligible to participate in the tutored internship but did not.

The framework generally used in evaluation analysis for this problem is the Roy-Rubin model [28, 29]. In case of a binary treatment, individuals have one of two mutually exclusive states. The ‘0’ stands for the untreated state and ‘1’ for the treated state, where *D* = 0 denotes non-treatment and *D* = 1 denotes treatment. An outcome is associated with each state. The potential outcome is expressed as *Y*_0_ for untreated state and *Y*_1_ for treated state. This outcome can be earnings, being employed, passing a competition, etc. It is generally the variable of interest for which the programme was implemented. In our study, the outcome is the propensity of an individual to work with FA, expressed as a percentage of hours worked in FAS. We used the most common evaluation parameter of interest, which is the average treatment effect on the treated (ATT) where:

$$ATT=E(Y_{1}-Y_{0}|D=1)=E(Y_{1}|D=1)-E(Y_{0}|D=1)$$ (1)

The ATT answers the question: ‘How much did veterinary students participating in the tutored internship programme work in FAS after they graduated compared to what they would have worked without participating in the programme?’ Thus, it estimates the realized gross gain from the treatment. *E*(*Y*_1_|*D* = 1) can be computed because it only requires to compute the outcome of the treated. The issue is to find the counterfactual mean *E*(*Y*_0_|*D* = 1) that is not observed. Indeed, data on non-participants enable one to identify only *E*(*Y*_0_|*D* = 0). So, one has to select a relevant substitute for *E*(*Y*_0_|*D* = 1) in order to estimate ATT.

#### 2.2.2 Selection on observables

The solution advanced by Rosenbaum and Rubin [30] is based on the assumption that given a set of observable covariates X, potential outcome of non treated *Y*_0_ is independent of participation status. This is called the *conditional independence assumption* (CIA) (or *selection on observables assumption*):

$$Y_{1},Y_{0⊥}D|X.$$ (2)

Hence, conditionally on X, the mean of the potential outcome is the same for *D* = 0 and *D* = 1. It means that:

$$E(Y_{0}|D=1,X)=E(Y_{0}|D=0,X)$$ (3)

The assumption (2) has empirical content only if there are participants and non-participants for each observable characteristics X. Formally, it assumes that:

0<Pr(D=1|X)<1. (4)

To satisfy the assumption (4), known as *overlap condition*, there must be at least one participant and one non-participant for each X.

If we are interested in estimating the ATT only, the conditions (2) and (4) can be weakened. Thus one needs only to assume:

$$Y_{0⊥}D|X,$$ (5)

And

Pr(D=1|X)<1 (6)

The assumptions (5) and (6) are sufficient to identify the ATT. Hence, according to (1) and (3), ATT can be written as follows:

$$ATT=E(Y_{1}|D=1,X)-E(Y_{0}|D=0,X).$$ (7)

Hence, the average treatment effect is the expected difference in outcome between treated and untreated units with respect to X.

We also used the quantile treatment effect on the treated (QTT) to know the distributional effect of the treatment, beyond the average effect. To identify QTT, one has to verify the *rank preservation assumption* [31, 32] which requires the relative rank of the potential outcome for a given individual to be the same, regardless of treatment status. Moreover, quantiles must be well defined and unique for each *τ in* (0,1) [33]. The latter assumption is known as the *existence and uniqueness assumption*. Under the *rank preservation assumption*, *existence and uniqueness assumption*, (5) and (6), QTT become identified and estimable and can be expressed as follows:

$$QTT=q_{\tau }(Y_{1}|D=1,X)-q_{\tau }(Y_{0}|D=0,X)$$ (8)

Where *τ* is a real parameter in (0,1) that reflects a percentile and *q*_*τ*_ is the *τ*-th quantile of the outcome *Y*_*j*_ (*j* = 0, 1)conditional on treatment D and covariates X.

#### 2.2.3 Matching methods based on the propensity score

The main purpose of matching methods, under assumptions (5) and (6), consists of comparing the outcomes of treated and untreated units with ‘similar’ conditioning on observable characteristics (as age, gender, education…). This ‘similarity’ can be made using a distance such as Euclidean distance or the Mahalanobis distance or using a balancing score, such as the propensity score. Instead of conditioning on X, Rosenbaum and Rubin suggest conditioning on a propensity score (PS) [30]. The propensity score is defined as the probability of participation for individual *i* given a set X of characteristics where *p*(*X*) = *Pr*(*D* = 1|*X* = *x*_*i*_). If (4) and (5) hold, then:

$$Y_{0}⊥D|p(X)$$ (3)

This assumption implies that in order to build the counterfactual conditional mean *E*(*Y*_0_|*D* = 1, *X*), we require to compute *E*(*Y*_0_|*D* = 1, *p*(*X*)) which is equal to *E*(*Y*_0_|*p*(*X*), *D* = 0).

The propensity score is obtained by estimating a logit model where participation in the tutored internship is regressed on relevant and correlated variables: age, gender, if the student comes from a rural background, whether the student reoriented themselves towards a predominantly rural sector when they were in an NVS, and work status of the parents. For each matching method, we added a condition that consists of matching a treated veterinary student with a control who graduated the same year, in order to harmonize the treatment effect.

Once the propensity score is evaluated for each unit, a matching algorithm must be chosen to match the treatment group with the control group from their propensity score. The most widely used matching algorithms after propensity score evaluation are Nearest-Neighbour (NN) Matching, Radius or Caliper Matching, Kernel Matching and Stratification Matching [34]. In this study, we will use nearest neighbour matching within caliper (NNC), a combination of NN and Caliper matching.

Since we have 101 treated and 171 untreated individuals, some treated could be matched to more than one untreated. Therefore, several matchings (1:1, 1:2 and 1:3) will be tried and the best method in terms of balance retained.

*2*.*2*.*3*.*1 Nearest neighbour matching within Caliper (NNC)*. The aim of this method is to randomly order treated and untreated units. Then, it selects a treated unit *i* and finds a match *j* in the control group if this absolute difference is the smallest among all pairs of absolute differences of PS between *i* and others *j*s.

The neighbourhood *C*(*P*_*i*_) of a treated unit *i* is defined as:

$$C(P_{i})=\underset{j}{\min}\left|P_{i}-P_{j}\right|,j\epsilon I_{0}$$

Where *P*_*i*_ and *P*_*j*_ are the propensity score for treated and control participants, *I*_1_ and *I*_0_ the set of treated and control participants, respectively. For each treated unit *i*, one or several unit *j* can fall into *C*(*P*_*i*_). The issue is that the distance between *P*_*i*_ and *P*_*j*_ is not restricted. Hence a very different unit *j* can match a treated unit *i*. To avoid this shortcoming, another condition must be imposed by selecting a caliper *ε*:

$${|P}_{i}-P_{j}|<\varepsilon ,j\epsilon I_{0}$$

The caliper width defines the range where the propensity scores must fall to be considered a valid match. Therefore, according to NNC method, a unit *i* matches a control unit *j* if: first, the absolute difference is the smallest among all pairs of absolute differences of PS between *i* and others *j*s; and second, if the absolute difference of propensity score between *i* and *j* falls into a caliper *ε* [35]. If units *i* and *j* are matched, they are removed and the next treated participant is selected. From a study that compared matching methods using different calipers using Monte Carlo simulations to find the optimal caliper, the threshold of 0.2 is recommended [36].

#### 2.2.4 Matching methods without propensity score

Other matching methods, which do not use propensity score, are used in this study to measure the effect of the programme.

*2*.*2*.*4*.*1 Mahalanobis distance Matching (MDM) without PS*. MDM takes each treated unit and matches it to the nearest control unit with Mahalanobis distance, which is defined by [37]:

$$md(X_{i},X_{j}){=\{{{(X}_{i}-X_{j})}^{T}S^{-1}{(X}_{i}-X_{j})\}}^{\frac{1}{2}}$$

Where X denotes the matrix of covariates, *i* and *j* treat and control units and S the covariance matrix of matching variables X. The control unit *j* with the minimum distance *md*(*X*_*i*_, *X*_*j*_) is selected as the match for treated participant *i* and both participants are removed from the pool. This process is repeated until all treated units are matched. An estimated propensity score could be included in the covariates. If this is the case, the method is called MDM with PS; otherwise, it is called MDM without PS [35]. In this study, we focus only on MDM without propensity score.

*2*.*2*.*4*.*2 Coarsened Exact matching (CEM)*. The CEM algorithm works in three steps. First, each continuous covariate is coarsened into natural breakpoints (as quartiles) and transformed into categorical variables. Discrete variables are left as they are or combined. Second, all units that have the same values of the coarsened variables are sorted into bins. Third, all the units that are in bins that do not include at least one treated unit are deleted. After constructing and filling the bins, all units are weighted. Unmatched units get a weight of 0, whereas matched units in the treatment group get a weight of 1. Matched units in the control group get positive weights that normalize the bins. The formula of the units’ weights in a bin *b* is: ${weights}_{b}=\frac{\frac{Number\;of\;treatments\;in\;the\;bin}{Number\;of\;controls\;in\;the\;bin}}{\frac{Total\;of\;matched\;control}{Total\;of\;matched\;treated}}$

#### 2.2.5 Covariate balance diagnosis after matching

The advantage of matching methods is to obtain a control group with the same distribution of characteristics as the treatment group. The distribution of these covariates should be similar across levels of the treatment to be balanced. The standardized mean difference (SMD) provides a relevant view of covariate balance [38] and will be computed for each covariate before and after matching.

#### 2.2.6 Covariate selection

According to the theory of causal inference [39], covariates that have causal effects on the outcome, whether or not they have causal effects on the treatment, should be included in the matching models, particularly in small samples as in this study. However, among these covariates, some should be correlated to the treatment to properly estimate the propensity score. Variables that are correlated to the treatment but not to the outcome must be excluded, otherwise they can strongly decrease the precision of programme estimation [40]. Moreover, economic theory and knowledge about the contextual setting should guide the researcher in building the model [41, 42].

## 3. Results

### 3.1 Descriptive statistics

**Fig 1:**
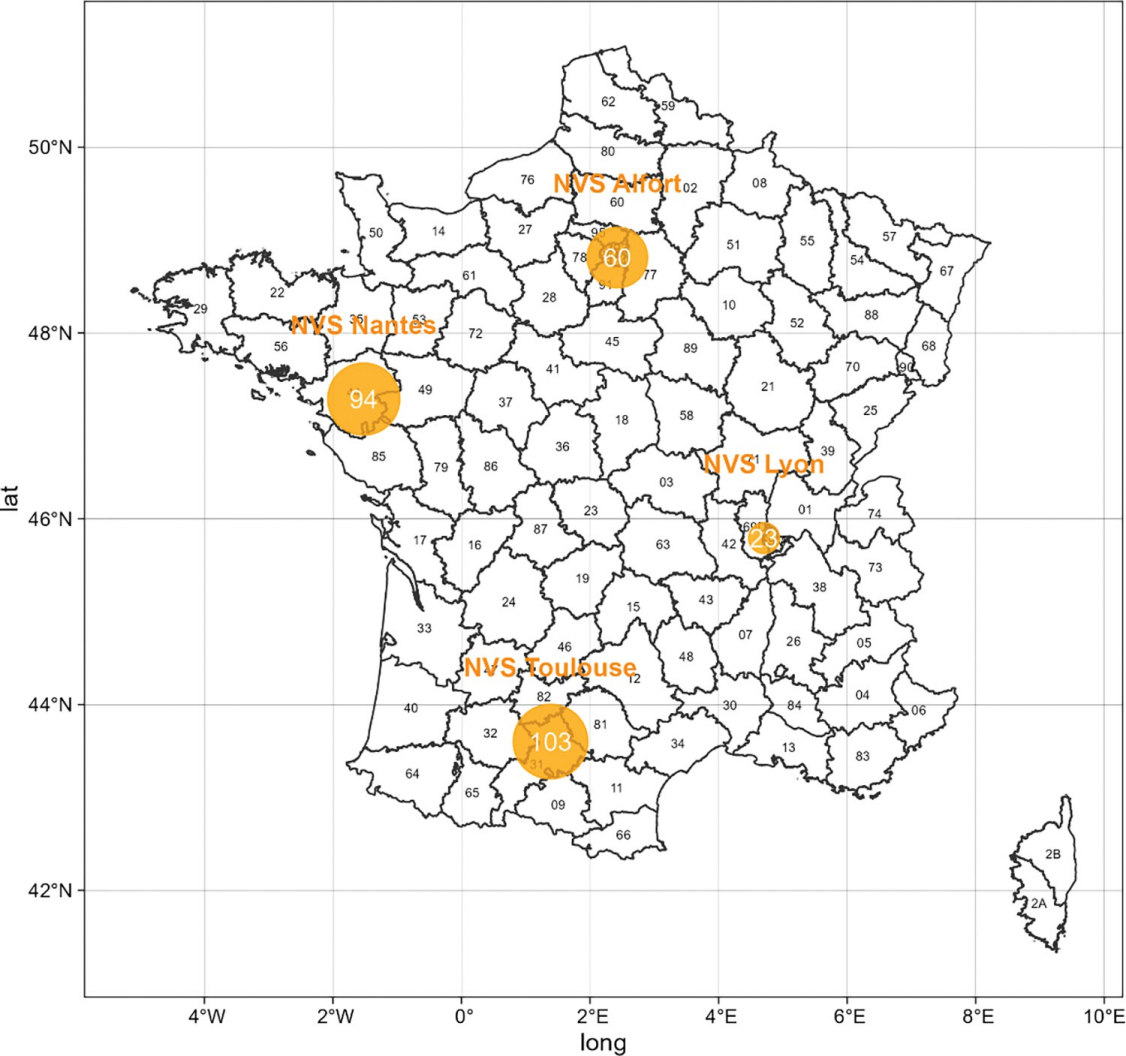
Distribution of respondents to the questionnaire. Source: author. Generated by R software (version 4.3.1) and the packages sf and ggplot2.

In total, 280 veterinarians from the 4 NVS who completed their final year with FA focus responded to the survey. We retrieved answers from approximately 50% of the total population surveyed (550 questionnaires sent). One hundred and one respondents completed a tutored course, which constitutes 57.4% of the total student population who completed a tutored internship between 2016 and 2020. One hundred and seventy-nine respondents did not complete a tutored internship, representing 48.7% of the total student population in the final year with FA focus that did not complete a tutored internship in the same period. The distribution of responses by NVS are in Fig 1. In each school, we interviewed veterinarians who had completed a tutored internship and those who had pursued a final year with FA focus without tutored internship, with the exception of the NVS Lyon, where we were only able to contact veterinarians who had completed a tutored internship but not potential control units.

Overall, 67.5% of respondents were women (Table 1). Moreover, 51.4% of veterinary students have rural backgrounds. Socio-professional category (SPC) of the respondents reveals that 11.64% of them have a parent who is a farmer and 14.5% of them have a parent who is in the ‘working class’ which is composed of ‘employee’ (10.9%) and ‘worker’ (3.6%) professional categories according to the French nomenclature of professions and socioprofessional categories made by the Institut national de la statistique et des études économiques (INSEE). Of the respondents, 19.3% reoriented themselves towards FAS during their course at the NVS. The veterinarians interviewed represent 36.7, 33.5, 21.4 and 8.2% of NVS Toulouse, Nantes, Alfort and Lyon, respectively.

**Table 1:** Descriptive statistics on survey data.

Participation in a tutored internship (treatment in %)	Non treated	64
	Treated	36
Share of work in food animal sector (outcome)	mean	52.16
	std	32.6
	min	0
	25%	30
	50%	50
	75%	80
	max	100
Gender (%)	Men	32.5
	Women	67.5
Background (%)	Rural	51.4
	Urban (< 10,000 inhabitants)	21.7
	Urban (> 10,000 inhabitants)	28
Socio-professional category (%)	Farmers	11.64
	Craftsmen, merchants, company managers	12.73
	executives and higher intellectual professions	43.64
	Employees	10.91
	Workers	3.64
	Intermediate professions	12.36
	Retired	4.36
	No professional activity	0.73
Reorientation during the school career (%)	Food animals	19.3
	Mixed sector (food animal and companion animals)	17.9
	No reorientation	62.8
Age	mean	28
	std	1.73
	min	25
	25%	27
	50%	28
	75%	29
	max	34
National Veterinary School (%)	NVS Toulouse	36.7
	NVS Alfort	21.4
	NVS Lyon	8.2
	NVS Nantes	33.5

Source: Survey data

### 3.2 Testing for balance

Sample differences before and after matching are in Table 2. The average age of veterinarians in treatment and control groups was 27.91 and 28.06 years, respectively. The treatment group was composed of 69% of women compared to 66% in the control group. Additionally, 46% of treated veterinarians were from a rural background whereas 51% of controlled veterinarians were from an urban background. Treated are more likely to have a parent who is employee or worker (20 *vs* 12%) and are more likely to have a parent who is farmer (13 *vs* 10%). The percentage of treated who reoriented to FAS during their course at the NVS is smaller than the percentage of controlled (16 *vs* 21%).

**Table 2:** Univariate statistics before and after matching for covariates.

		Before matching			After matching		
**NNC**	covariates	treated	control	SMD	treated	control	SMD
	age	27.91	28.06	0.08	27.94	27.72	0.12
	woman (%)	0.69	0.66	0.06	0.7	0.72	0.03
	rural origin (%)	0.46	0.51	0.1	0.44	0.44	0.01
	parent farmer (%)	0.13	0.1	0.08	0.12	0.1	0.08
	parent employee/worker (%)	0.2	0.12	0.2	0.13	0.11	0.06
	reoriented to rural (%)	0.16	0.21	0.15	0.17	0.14	0.06
	N observations	101	179	NA	90	151	NA
**MDM**	age	27.91	28.06	0.08	27.91	27.76	0.08
	woman (%)	0.69	0.66	0.06	0.69	0.7	0.02
	rural origin (%)	0.47	0.51	0.1	0.47	0.5	0.08
	parent farmer (%)	0.13	0.1	0.08	0.13	0.11	0.06
	parent employee/worker (%)	0.2	0.12	0.2	0.2	0.13	0.17
	reoriented to rural (%)	0.16	0.21	0.15	0.16	0.16	0
	N observations	101	179	NA	101	101	NA
**CEM**	age	27.91	28.06	0.08	27.91	27.7	0.1
	woman (%)	0.69	0.66	0.06	0.72	0.72	0
	rural origin (%)	0.47	0.51	0.1	0.46	0.46	0
	parent farmer (%)	0.13	0.1	0.08	0.12	0.12	0
	parent employee/worker (%)	0.2	0.12	0.2	0.17	0.17	0
	reoriented to rural (%)	0.16	0.21	0.15	0.12	0.12	0
	N observations	101	179	NA	87	123	NA

Source: Survey data

**Fig 2:**
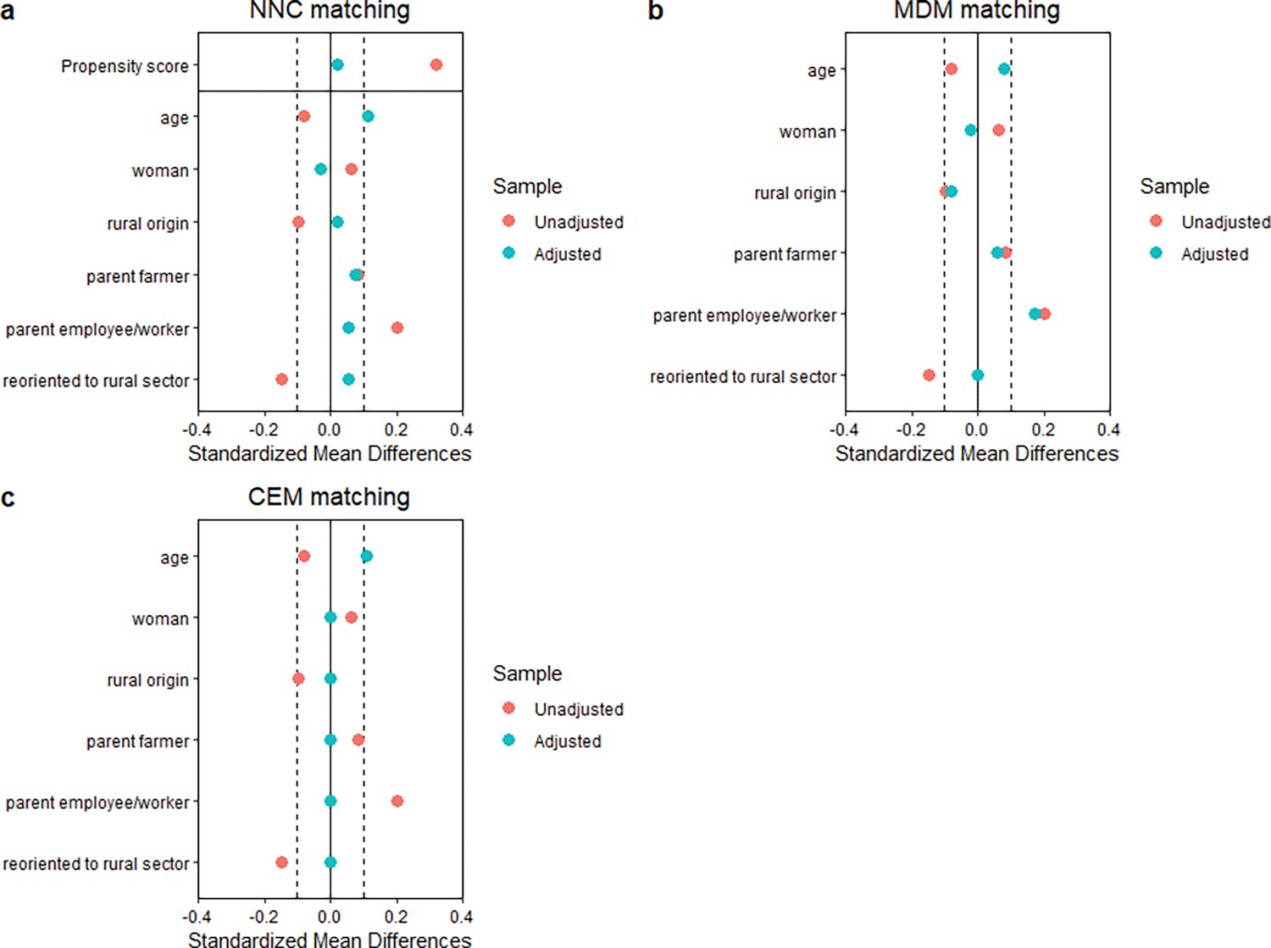
Covariate balance diagnosis. Source: Survey data.

A comparison of the covariate balance before and after matching for each method to check whether balance has improved is shown (Fig 2). The NNC matching method improved all covariate balance except variable *age*. Additionally, in the MDM matching method the SMD of all variables decreased except the SMD of the *age* that kept the same value. The CEM has an excellent balance since the SMD of all the variables is equal to 0 except for covariate *age* that is less balanced after the matching. Interestingly, the matching methods do not have the same number of unmatched treated units. NNC, MDM and CEM pruned 11, 0 and 14 treated units, respectively.

### 3.3 Estimation of the treatment effect

Results of the NNC, MDM and CEM methods (Table 3) suggest an average difference in share of work in FAS (ATT) between -0.57 and 1.64% that is not statistically significant. Therefore, the ATT does not provide any evidence of a positive average effect of the tutored internship on the veterinarians’ share of work in FAS.

**Table 3:** Tutored internship effect according to each method.

	NNC		MDM		CEM	
	Coefficient	Standard error	Coefficient	Standard error	Coefficient	Standard error
ATT	1.644	4.31	-0.574	4.365	1.257	4.83
QTT						
0.1	0	4.919	1.1	7.446	0	4.905
0.2	13.58*	8.134	10.333	9.253	20**	9.766
0.3	9.786	8.552	2.014	8.548	9.321	8.095
0.4	0.429	6.351	-0.081	4.753	0.255	6.833
0.5	0.071	4.731	-0.267	4.727	0.364	3.819
0.6	1.929	7.527	-1.2	7.942	-0.214	6.244
0.7	-4.75	6.618	-8.089	7.958	2	6.958
0.8	-8	6.412	-9.733	6.066	-7.5	7.48
0.9	-5.737	5.085	-3.417	5.171	-7.743	6.153

Note: Bootstrapped standard errors for QTT estimation are based on 500 replications. In all methods, an exact matching constraint on the year of graduation was performed, i.e., every treated veterinarian was matched with a veterinarian in the control group that graduated in the same year.

*p< 0.1

** p < 0.05.

Source: Survey data

Regarding QTT estimates, positive and significant estimates are present for the second decile which are 13.58% (p-value <0.1) and 20% (p-value <0.05) for NNC and CEM matching, respectively. Hence, the tutored internship has a more pronounced effect for veterinarians who have a lower work percentage in FAS.

## 4. Discussion

This study evaluates effects of veterinary students’ tutored internship on their percentage of work in the food animal sector (FAS). To that end, we conducted a survey and we used three matching methods to assess impacts of the tutored internship.

Evaluating a public policy consists of finding out whether the legal, administrative or financial means implemented have achieved objectives set for it in order to improve public policy and better orient it. Many econometric methods have been developed to address the issue of causal effect identification. The most direct method is controlled experiments, which consists of randomly selecting people who will and people who will not benefit from a programme [43]. This method cannot be used in our case because we assess a policy ex-post. This study used what is known as natural experiments (or nonexperimental), where the framework of controlled experiments is reproduced after the policy. Nonexperimental evaluation methods are relevant alternatives to experimental designs but they rely on some untestable assumption [30]. The classical nonexperimental methods are difference-in-difference, instrumental variable, regression on discontinuities, and selection methods on observables [43]. In many cases, it is challenging to obtain all the required data for the first three methods. Indeed, in the difference-in-differences method, the evolution of the situation of beneficiaries before and after implementation of the programme is compared to that of a group of non-beneficiaries, assuming that the evolution of the two groups is similar. However, this method cannot be used in our study because the outcome in this study is the propensity of activities in FAS and can’t be observed before the treatment, because all veterinarians started working after the treatment. The instrumental variables method relies on existence of factors that affect the probability of participating in a programme but not the unobserved control variables. In our case, we did not identify any variable that can perform such a function. The third method is regression on discontinuities. This method is based on a continuous (or discrete) selection variable. The idea is to exploit the thresholds of assignment in certain selections to a treatment. For example, exploit the grade obtained in the baccalaureate as a condition to receive study grants. The principle of this method is that individuals who have obtained a mark slightly below this threshold and who do not receive this study grant are almost identical to individuals who are slightly above the threshold and who receive this study grant. In our study, there is no such continuous variable that selects the veterinarians who will participate in the tutored internship.

When none of these methods verify the condition of application, the selection effect can be corrected by controlling for observable differences between treated and untreated units using matching methods. There are two major classes of matching methods. The first is the largest class of matching methods known as the Equal Percent Bias Reducing (EPBR) that includes the Mahalanobis Distance Matching (MDM) and the Propensity Score Matching (PSM). The second class is the Monotonic Imbalance Bounding (MIB) where the leading example is the Coarsened Exact Matching (CEM). According to Iacus, King and Porro, MIB class improve significantly inferences relative to EPBR class [44].

The PSM was used via NNC method because it is the most popular matching method for its ease to be implemented in studies evaluating social programs and market policies [45, 46]. Other matching methods that do not use propensity score are recommended to test stability and robustness of the findings [47]. For example, Mahalanobis Distance Matching (MDM) without propensity score matching and Coarsened Exact Matching (CEM) are two relevant alternatives [35, 48, 49]. The advantage of MDM and CEM is that they approximate a fully blocked randomized experiment, whereas PSM attempts to approximate a completely randomized experiment. Fully blocked experiments have better standards because they have less imbalance, less model dependence, less bias and more power, more efficiency and more robustness than completely randomized experiments [49]. Moreover, MDM and CEM guarantee that samples are well-balanced overall but also guarantee that the paired units are close, whereas PSM balance samples but sometimes with not close pairs [49]. All in all, there is no ‘best’ matching method in absolute terms. Generally, matching is done in several ways and the method that provides the best balance in the observed characteristics between treated and control groups is selected [47, 50, 51].

The observed characteristics described in Table 1 indicate an overrepresentation of women (67.5%) among young veterinarians. This is consistent with feminization of the veterinary profession where women represent 73.9% of the population of veterinarians under 40 years of age [1, 52]. Concerning the background of the veterinarians, 50.3% come from a rural environment, quite similar to a survey conducted in NVS of Toulouse indicating that 54% of veterinarians who did a fifth year with a FA focus, as respondents to our survey, had a rural background [53]. Furthermore, regarding the socio-professional category of respondents, 11.64% have a parent who is a farmer, much larger than the percentage of farmers in the French population, which is less than 2% [54]. *Executives and higher intellectual professions* and *employees* categories are also significantly different from national statistics (43.64 and 10.91% in the survey compared to 18.7 and 41.1% at the national level [54]). All covariates were collected in a survey carried out in collaboration with veterinarians who had chosen a fifth year with a FA focus. Among them are those who have followed a tutored internship and those who have not.

Particular attention must be paid in a situation of nonrandom selection because the decision to participate in a programme may be endogenous with respect to outcomes. In this study, we assumed that the non-random selection does not bias treatment effect estimation because the decision to participate in the programme is based on several factors not related to the outcome (share of work in FAS). First, according to the above mentioned survey [53], students in the fifth year with FA focus make a trade-off between having more classes and not participating in the tutored internship–because most students will not have the opportunity to have specialised courses after graduation–and practising in the field by participating in the tutored internship. Second, some students prefer to be mentored by an experienced veterinarian at the beginning of their career to improve their relationship with clients in rural areas, which geneally requires more experience than clients in the companion animal sector [53]. Third, a factor that could have been decisive in the choice of the students is the economic factor. Indeed, students could have chosen to do a tutored internship simply to get paid. However, based on the above-mentioned survey, among 12 factors that were proposed to students who did a tutored internship, the financial factor was the least important. This allows us to assume that participation in the tutored internship is not endogenous regarding outcomes.

To assess effects of the tutored internship on the tutored veterinarians’ rural practice, respondents were asked about their share of work in FAS, which represents the outcome. This questionnaire was conducted among veterinarians who graduated between 2016 and 2020. As the survey was conducted in November 2022, the outcome variable is not homogeneous among all participants. This is because the outcome of a veterinarian who graduated in 2016 cannot be considered equivalent to that of a veterinarian who graduated in 2020. To overcome this issue, we matched control and treatment groups by adding a condition to the matching that consists of imposing to each treated individual who graduated in year *n* an untreated individual who graduated in the same year.

The three matching methods enable to balance treatment and control groups. According to the SMD, the three matching methods improve balance for all covariates except *age*. Additionally, in all methods, the absolute value of SMD of all covariates are < 0.1 except for the variable *parent employee/worker* in MDM and covariate *age* in NNC that have a SMD of 0.17 and 0.12 after matching, respectively, which indicates that matches were successfully completed. It is generally endorsed that an absolute value of SMD <0.1 is a reasonable cut-off for acceptable SMD [55]. CEM, NNC and MDM pruned 14, 11 and 0 treated units, respectively. Matching methods usually prune observations from a dataset to reduce imbalance between treatment and control groups. Pruned observations may reduce the variance of the causal effect estimate but may also inflate this variance if too many observations are pruned which leads us to a bias-variance trade-off. Since matching methods do not optimize with respect to imbalance and matched sample size simultaneously, we used three matching methods to identify a good matching solution. The pruned treated units in CEM and NNC methods can also be due to the additional condition that consists in associating a treated veterinarian with a controlled veterinarian who graduated the same year, which reduces the candidates in the control group that could be matched with treated units. The optimal balance of CEM is consistent with the fact that CEM dominates commonly other matching methods [48]. CEM has the advantage of having a balance between covariates, but MDM has the advantage of reducing the number of unmatched treated units.

The results of the NNC, MDM and CEM matching methods (Table 3), suggest an average difference in share of work in FAS (ATT) which is statistically non-significant. Using various matching methods and obtaining converging results reinforce our confidence in our interpretation of the results. The absence of a significant effect on average of the treatment suggests that the tutored internship does not increase the likelihood to practise most of the time in the FAS, and does not address the problems of barriers that prevent veterinarians to settle in rural areas. There are a whole range of reasons that could explain why veterinarians do not want to practise in rural areas.

First, food animals are generally located in remote areas, which can limit access to various amenities, but also limit access to work for the other partner of the household.

Second, in our survey, we asked veterinarians who received an offer from the veterinary clinic after being in the programme and who turned down the position. Most participants mentioned the fact that they had to join their partner.

Third, the veterinary clinic offers a sustained workload and is often exclusively oriented towards rural activities according to the survey. To some extent, the training received during the tutored internship enables the fifth year students to discover the reality of practice, but does not address any of these three issues. Overall, the lack of attractiveness of rural areas affects all young veterinarians, regardless of their background.

Noteworthy, based on QTT results, the tutored internship still has a positive and significant effect between 13.6% (p-value <0.1 according to PSM) and 20% (p-value < 0.05 according to CEM) for veterinarians who are under the second decile of the outcome. This suggests that enrolling in a tutored internship is associated with an increased probability of working a limited amount of time with FAS. According to our survey, 8% of veterinarians who have completed a tutored internship do not work in FAS—consistent with results of a previously cited survey [53]—whereas this figure rises to 16% for veterinarians who have not completed a tutored internship. This shows that the tutored internship incites some veterinarians to develop a network in the rural environment, and encourages them to maintain an activity, albeit reduced, in the FAS.

To our knowledge, this is the first study evaluating the effectiveness of programmes that aim at encouraging veterinarians to work in FAS. We can still draw arguments from the literature in human medicine, where interest in the shortage of physicians in rural areas represents a similar situation. Several authors evaluated effects of retention programs and incentives schemes of general practitioners in rural areas [19, 56–58]. According to these authors, it is clear that the effects of incentives for medical students to practice in rural areas is not well established and more studies are needed.

However, based on the implication of literature, the mechanisms behind our results can be explained by the fact that there is no single measure which is likely to improve substantially health workforce in rural areas. The measures that emerged from the literature are to maintain an adequate infrastructure [59] and staffing [60], to guarantee a competitive remuneration [57], to reward individuals making a significant contribution and to ensure an attractive and stable environment for individuals’ family [61] which is an important aspect of work-life balance. Another survey [62] showed that the most important working conditions for students interested in FAS are, in order of preference, the opportunity to practice high-quality medicine, to have an employer fair in assignment of cases, the opportunity to keep up skills in small animal medicine and then an adequate salary. While veterinary graduates recently have been more interested in working hous, salary and paraprofessional activites. Additionally, most of strategies suggest to bundle retention incentives, which has not been targeted for the tutored internship.

Development of appropriate strategies requires a preliminary understanding of factors influencing veterinarians willingness to work in rural areas and in FAS [19]. First, a discrete choice experiment showed that there were some significant incentives to retain physicians in rural areas as financial incentives and a job located close to respondents’ home [63]. But non-pecuniary incentives seem to have stronger effects than financial incentives [64] for general practitioners and a targeted approach in incentive design is recommended [65]. In the case of veterinarian shortage, one recommendation could be to offer to veterinarians who have a rural background—more inclined to settle in rural areas- the best conditions to work and offer them better incentives tailored to veterinarians’ characteristics to settle in or near rural areas. Second, a study provided evidence that general practitioners would be willing to give up 8.6% of annual personal earnings to work there while they require an incentive of at least 130% of personal earnings to go to the least attractive jobs [66]. It showed that the healthcare personnel shortage is not only a question of isolated location but also a matter of quality of work. Third, movement of general practitioners occurs when they are early-career [67], therefore it is more effective to target newly qualified veterinarians to provide them financial incentives than experienced veterinarians who are more stable [65]. Fourth public services seems to be a key issue for attracting veterinarians in rural reas [68]. All in all, difficulties to attract and retain veterinarians need bundles of incentives such as living environment, job conditions and career perspectives [19] and require structural changes rather than isolated public policies.

The results obtained in this study could be of importance for our understanding of the effects of public policies that aim at tackling veterinary shortage in rural areas. Despite the recommended sensitivity analysis to test the robustness of the results, four things should be kept in mind while interpreting these results. First, the propensity score was debated [69] and challenged in particular by King and Nielsen [49]. Regardless, their recommendations as setting a restrictive caliper and the evaluation of treatment and control imbalance have been followed. In this paper, we used a restricted caliper of 0.2 and particular attention was paid to the imbalance diagnosis by using the SMD. Moreover, two other methods, CEM and MDM, were used to corroborate our results. Second, to estimate a programme impact with selection on observables, one has to make some untestable assumptions. According to Heckman [25], the ideal way to estimate a programme impact is: (i) when treatment and control groups have the same distribution of observed characteristics (ii); and the same unobserved characteristics; (iii) when the same questionnaire is administered to both groups; and (iv) when treatment and control groups are in the same economic environment. The four conditions are filled except condition (ii) which is untestable. Third, the average treatment effect is not estimated in only 1 year but between 2016 and 2020. Therefore, we estimated the average treatment effect of tutored internship for veterinarians who graduated in different years. To overcome this limit, we matched only veterinarians who graduated in the same year. Fourth, results of this study should be interpreted with caution because there are no similar studies except studies on physician shortage.

## 5. Conclusion

This study evaluates effects of veterinary students’ tutored internship on their share of work in food animal sector (FAS) using three matching methods. If the tutored internship encourages veterinarians who have a low share of work in FAS to work a little more in this sector, it does not encourage veterinarians to have predominantly rural activity. Targeted strategies need to be developed to encourage veterinarians to work in FAS as recruiting early-career veterinarians from rural backgrounds, find a job location close to the veterinarians’ home, and increase the veterinarians’ quality of work in rural areas.

## Supporting information

S1 Table Survey. (PDF)
