# Supplementary material for: Effectiveness of rural internships for veterinary students to combat veterinary workforce shortages in rural areas
Source: PLoS One. 2024 Mar 7;19(3):e0294651. doi: 10.1371/journal.pone.0294651 (PMC10919651; doi:10.1371/journal.pone.0294651)
Supplement: S1 Table — (PDF) [file pone.0294651.s001.pdf]

Supplementary Materials

Supplementary Table 1: Survey

**Questions**
Gender
Age
What is your status (employee, partner, etc.)
Did you grow up in a rural / urban < 10,000 inhabitants / urban > 10,000 inhabitants environment?
Do you have a parent who is a farmer or in the agricultural field?
Did you have any experience in the agricultural field before entering the National Veterinary School? If yes, please indicate what this experience was.
What is the socio-professional category of your first parent?
What is the socio-professional category of your 2nd parent?
What activity did you want to do before entering one of the National Veterinary Schools?
Has this desired activity been modified during your studies?
If so, which activity did you change to?
What activity do you carry out today?

What is the proportion of rural activity in your job (in %)
If you are currently working in rural areas, what is the main production?
What is the travel time from your job to the nearest prefecture or sub-prefecture?
If you currently have a job, please indicate the postcode of your professional address.
Have you completed a tutored internship?
